# Overwintering States of the Pale Grass Blue Butterfly *Zizeeria maha* (Lepidoptera: Lycaenidae) at the Time of the Fukushima Nuclear Accident in March 2011

**DOI:** 10.3390/insects10110389

**Published:** 2019-11-04

**Authors:** Ko Sakauchi, Wataru Taira, Mariko Toki, Yuta Iraha, Joji M. Otaki

**Affiliations:** 1 The BCPH Unit of Molecular Physiology, Department of Chemistry, Biology and Marine Science, University of the Ryukyus, Okinawa 903-0213, Japan; kojikakoujika@yahoo.co.jp (K.S.); wataira@gmail.com (W.T.); 589kohaq@gmail.com (M.T.); iraha.s.1017@icloud.com (Y.I.); 2 Center for Research Advancement and Collaboration, University of the Ryukyus, Okinawa 903-0213, Japan

**Keywords:** developmental stage, field survey, Fukushima nuclear accident, larval instar, life history, lycaenid butterfly, overwintering, *Oxalis corniculata*, pale grass blue butterfly, *Zizeeria maha*

## Abstract

The Fukushima nuclear accident in March 2011 caused the massive release of anthropogenic radioactive materials from the Fukushima Dai-ichi Nuclear Power Plant to its surrounding environment. Its biological effects have been studied using the pale grass blue butterfly, *Zizeeria maha* (Lepidoptera: Lycaenidae), but the overwintering states of this butterfly remain elusive. Here, we conducted a series of field surveys in March 2018, March 2019, and April 2019 in Fukushima and its vicinity to clarify the overwintering states of this butterfly at the time of the Fukushima nuclear accident. We discovered overwintering individuals in situ associated with the host plant *Oxalis corniculata* under natural straw mulch as first-instar to fourth-instar larvae in March 2018 and 2019. No other developmental stages were found. The body length and width were reasonably correlated with the accumulated temperature. On the basis of a linear regression equation between body size and accumulated temperature, together with other data, we deduced that the pale grass blue butterfly occurred as fourth-instar larvae in Fukushima and its vicinity at the time of the accident. This study paves the way for subsequent dosimetric analyses that determine the radiation doses absorbed by the butterfly after the accident.

## 1. Introduction

Massive amounts of anthropogenic radioactive materials have been released into the environment from the Fukushima Dai-ichi Nuclear Power Plant (FDNPP); the accident occurred on 11 March 2011 [1,2,3,4,5,6], and the largest release occurred on 15 March 2011 [7,8,9]. Although the impacts of the Fukushima nuclear accident on humans are difficult to evaluate and have only been reported in a few studies [10,11], the biological impacts have been evaluated mostly in field-based surveys and dosimetric simulation analyses using various animals and plants [12,13,14,15,16,17,18,19,20,21,22,23,24,25,26,27,28,29,30,31,32,33,34,35,36]. Among them, insects have proved to be excellent systems. Radioactive cesium has been detected in many species of insects [37,38]. Gall-forming aphids in contaminated areas showed striking morphological abnormalities, such as duplication of the abdomen and legs [39,40]. An extensively studied insect in Fukushima research is the pale grass blue butterfly, *Zizeeria maha* (Lepidoptera: Lycaenidae). There is accumulated field and laboratory evidence that this butterfly has been affected considerably by the Fukushima nuclear accident [11,41,42,43,44,45,46,47].

The pale grass blue butterfly has been examined since the Fukushima nuclear accident in Fukushima and its vicinity, including seven representative localities with various distances from the FDNPP. The first-generation adults after the accident were collected on 13–18 May 2011 [41]. Thus, the first-generation individuals had been exposed for approximately two months until they reached the adult stage. Although the first-generation adults showed only mild morphological abnormalities, subsequent generations that were reared in the laboratory showed higher levels of morphological abnormalities and mortality [41]. Furthermore, the fourth- or fifth-generation adults caught on 18–21 September 2011 showed a higher morphological abnormality rate than the first-generation adults [41]. Subsequent internal and external exposure experiments conducted on this species [41,45,46,47] and other species (the cabbage white butterfly, *Pieris rapae*) [48] confirmed the vulnerability of these butterflies to radiation exposure in the field.

However, although it is important to discuss dose–response relationships in radiological studies, the doses of radiation absorbed by these butterflies are unknown, partly because precise information on their state in the field at the time of the Fukushima nuclear accident is lacking. As UNSCEAR (United Nations Scientific Committee on the Effects of Atomic Radiation) notes in its publication [49], the lack of data on the absorbed doses is an important drawback of the series of Fukushima studies using the pale grass blue butterfly from the standpoint of conventional radiation biology. More concretely, the relatively high mortality and abnormality rates of this butterfly in the filed surveys and laboratory experiments [41,42,43,44,45,46,47] may be perplexing for radiation biologists if absorbed doses were not very high. Dosimetric calculations may potentially solve this problem. Estimating the doses of radiation absorbed by free-living animals in the field is often difficult, and such simulations may rest heavily on the general knowledge of researchers regarding how animals move around in contaminated areas. Fortunately, overwintering insects do not move much, and we supposed that butterflies stayed at a single site, likely as larvae, at the time of the Fukushima nuclear accident. To calculate the absorbed doses as accurately as possible, it is critical to know how larvae or other stages of this butterfly overwinter. Especially important is how large they were and how fast they grew. Without such information, accurate dosimetric evaluations of the radiation sensitivity of this butterfly are not possible.

The pale grass blue butterfly is a small lycaenid butterfly that is highly common throughout Japan (except Hokkaido). Due to this fact, its life history is relatively well known among Japanese lepidopterists [50,51,52,53,54,55,56,57]. The butterfly has the typical immature stages of holometabolous insects: egg, larva (first to fourth or fifth instars), prepupa, pupa, and adult. The life history depends entirely on the host plant, *Oxalis corniculata*, which is a common small weed called the creeping wood sorrel. The larvae of this butterfly consume this plant almost exclusively. Although there seems to be a consensus that the overwintering state of the pale grass blue butterfly is dormancy and not diapause, there is no solid consensus regarding the precise overwintering state of this butterfly in Japan. The Japanese lepidopterological literature mentions that this species overwinters mainly as third-instar and fourth-instar larvae [50,51,52,53], but it was also mentioned that there are no specific overwintering stages in this butterfly [50,54]. One regional report described that all life stages from eggs to adults were observed in December 1964 in Kuno, Shizuoka City, despite the abundance of third-instar and fourth-instar larvae, and that fourth-instar larvae were almost exclusively observed in January and March in 1964–1966 in other regions of Shizuoka Prefecture [50]. The striking difference between Kuno and other regions in Shizuoka Prefecture has been attributed to a climatic difference; Kuno is an exceptionally warm region [50]. However, to the best of our knowledge, there has been no systematic ecophysiological study of the overwintering states of this butterfly in Japan, not to mention in Fukushima. Two books that specifically focus on butterflies in Fukushima do not provide any useful information on the overwintering states of the pale grass blue butterfly [55,56].

In this study, we investigated how this butterfly overwinters in the field in Fukushima and its vicinity. We carried out three rounds of field work in March 2018, March 2019, and April 2019, and we successfully found overwintering immature stages of this butterfly in situ. In this sense, this study contributes to the lepidopterological life-history knowledge of this butterfly. Moreover, based on the field data, we endeavored to simulate the overwintering states of the butterfly in 2011. Using in situ larval size data and temperature records of 2019, we obtained a numerical ecophysiological relationship between larval size and temperature, which may be generally applicable to larvae of previous years in this region. On the basis of this relationship and the temperature records of 2011, together with other data, we quantitatively estimated the larval size at the time of the Fukushima nuclear accident in March 2011. Throughout this study, we tried to minimize the number of assumptions for size simulations, although some of them were required for calculations. As a result, this study built a solid foundation for dosimetric simulations of the doses of radiation absorbed by this butterfly. Eventually, we will be able to link field absorbed doses to toxicological data of this butterfly.

## 2. Materials and Methods

### 2.1. Butterfly, its Host Plant, and the Overwintering Period

**Figure 1:**
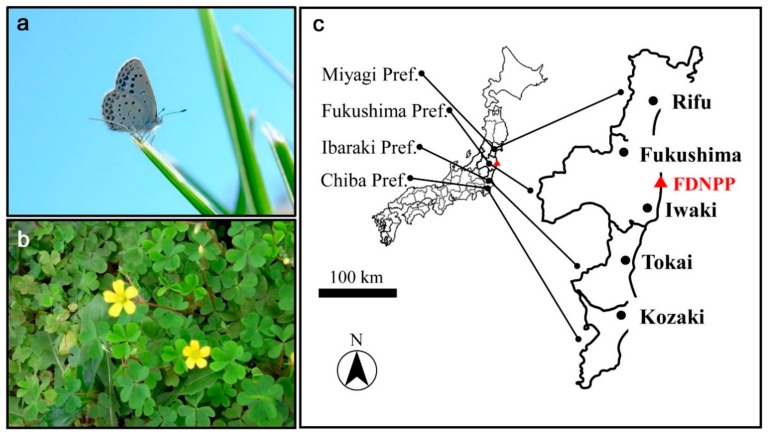
Organisms of interest and field work localities. (**a**) An adult individual of the pale grass blue butterfly, *Z. maha*; (**b**) The host plant, the creeping wood sorrel, *O. corniculata*; (**c**) Field work localities. The Fukushima Dai-ichi Nuclear Power Plant (FDNPP) is shown in red.

The pale grass blue butterfly, *Z. maha* (Kollar, 1844), was the focus of this study (Figure 1a). The overwintering state in Fukushima and its vicinity was of interest in this study. No permissions were needed to study, collect, and rear this species in Japan, since this species is tightly associated with its host plant, *O. corniculata* (Figure 1b). For the sake of discussion, the overwintering period (“winter” for this butterfly) was defined as the period from late November to the very end of March in the study areas.

### 2.2. Previous Field Surveys in 2011–2013

Previous field surveys for adult butterflies were performed in seven representative localities twice a year (in spring and fall) in 2011–2013 [41,44]. These localities (i.e., Fukushima, Motomiya, Hirono, Iwaki, Takahagi, Mito, and Tsukuba) are located at various distances from the FDNPP. The present study aimed to clarify the overwintering states of the pale grass blue butterfly at these seven localities in March 2011 based on data obtained in 2018 and 2019. Among the seven representative localities, Fukushima and Iwaki were also surveyed in the present study. The morphological abnormalities of this butterfly subsided by the end of 2013 [44]. Thus, the effects of the Fukushima nuclear accident on the overwintering states were likely minimal in 2018 and 2019.

### 2.3. Field Surveys in 2018 and 2019

In the present study, we conducted field surveys in Fukushima and Iwaki, which were two of the seven representative localities that were surveyed in 2011–2013 [41,44], as mentioned above (Figure 1c). Our choice of these two localities was based on information from our previous field work showing that the host plant was found abundantly in clusters, together with flying adult butterflies, there in summer. This means that the host plant (and thus larvae and other immature stages that may be associated with it) may be discoverable even in winter. In the other five localities among the representatives, the host plant was found to be scattered and less abundant in summer, which may make it difficult to find the host plant in winter. The survey sites must also allow field workers to crouch down on the ground safely for 2 h. Thus, the March 2018 surveys were conducted in Fukushima and Iwaki but not in Motomiya, Hirono, Takahagi, Mito, and Tsukuba.

In addition to Fukushima and Iwaki, southern and northern localities that are located near but outside the seven representative localities and that can be used as substitutes for the five other representative localities were considered for the March and April 2019 surveys. The candidate sites were ideally open fields near water (typical habitat of the host plant), which were provisionally identified using Google Maps. We made preliminary visits to Tokai and Kozaki and the other candidate localities except for Rifu in summer (September 2018) to check the abundance of the host plant and for flying adult butterflies. All together, we conducted field surveys in the following five localities (ordered from north to south): Rifu Town (Miyagi Prefecture), Fukushima City (Fukushima Prefecture), Iwaki City (Fukushima Prefecture), Tokai-Mura Village (Ibaraki Prefecture), and Kozaki Town (Chiba Prefecture) (Figure 1c).

We carried out three rounds of field surveys in total in Rifu (10 March 2019 and 10 April 2019), Fukushima (10 March 2018, 12 March 2019, and 11 April 2019), Iwaki (12 March 2018, 9 March 2019, and 12 April 2019), Tokai (12 March 2019), and Kozaki (13 March 2019 and 13 April 2019) in March 2018, March 2019, and April 2019. Field work at a given site was conducted for 2 h at maximum. At a site, after a habitat-wide observation, two or three field workers who were familiar with all the life stages of this butterfly and its host plant visually searched primarily for larvae, prepupae, and pupae but also for eggs and adults. Eggs are the smallest among the stages and instars, but they are relatively easy to find because of their whiteness against the green leaves. In contrast, first-instar larvae are the most difficult to find because of their smallness and green body color against the green leaves. Prepupae and pupae are also difficult to find, although relatively large, because they are not always associated with the host plant as a result of larval wandering before prepupation. Probably for this reason, we were not able to find any individual after a 30-minute survey in Tokai on 13 April 2019. Thus, the April field survey in Tokai was halted.

At a site, any covering materials on the ground, mostly dried leaves of large weeds (i.e., natural straw mulch), were manually eliminated, and the living host plant leaves underneath were identified. After the identification of the host plant, the plant surface, including the lower side of leaves, was visually scanned. The ground and other materials near the plant were also visually scanned. When discovered, the larvae were carefully placed on a blank grid sheet and photographed. Then, they were released back into their original position, and the covering materials were replaced. In April 2019, to obtain information on the duration of the prepupal and pupal stages, the larvae from Fukushima, Iwaki, and Kozaki were placed in a tube (58.5 mm height × 14.0 mm inner diameter) and transported to the laboratory at a low temperature (below 10 °C) for rearing.

### 2.4. Body Size Measurements and Estimates of Larval Instars

The images of the larvae (*n* = 69), prepupa (*n* = 1), and pupa (*n* = 1) that were discovered in the field were analyzed using ImageJ (National Institute of Health, Bethesda, MD, USA). The longitudinal length from the head to the end of the body and the transverse width in the middle of the body of larvae, prepupa, and pupa were measured. Additionally, pupae that were obtained from rearing (*n* = 4; 2 reared pupae from Fukushima and 2 reared pupae from Kozaki) were also measured similarly. The larval length data were placed in numerical sequence in rank order, and the gap values between the nearest data points were obtained. The largest or the second-largest gaps were considered as the instar gaps, assuming that there were relatively large longitudinal size differences before and after a molt between larval instars. The validity of these gaps was evaluated in reference to our own rearing experiences. Using the larval length data (and also the larval width data) and the effective accumulated temperatures (from climate records of 2019), a linear regression line was obtained, assuming that a linear model is reasonable for their relationship.

In this study, the head capsule width measurements for instars [58] were not performed for the following reasons. First, the body length measurements were not error-prone in this study, because larvae in cold temperatures were mostly in a fixed size without any movement. The body length is the largest part of a larva, and thus relatively small errors may be introduced. In contrast, if measured, relatively large errors may be introduced in values of the head capsule width because of its smallness. Second, the head capsule of this butterfly larva is very small and embedded within the body underneath. Thus, the measurements require us to sacrifice and dissect larvae or to rear them to wait for molting. In our field surveys, these procedures were not practical, because we released larvae back to the original positions. Third, the head capsule width measurements also suffer from individual variation within a population [59].

### 2.5. The Lower Development Threshold and the Effective Accumulated Temperature

We first tried to apply the reported values of the Yokohama population of the pale grass blue butterfly based on a rearing experiment, although no precise data were presented: 11.6 °C for the lower development threshold (developmental zero) and 383.3 DD (degree days or day degrees) for the effective accumulated temperature of the entire course of development from eggs to adults [57]. However, these numbers did not yield realistic results that could explain the adult emergence dates in May 2011, for which we have solid information based on our field work [41]. This failure may indicate either that the laboratory rearing data are not necessarily applicable to the field data because other environmental factors may modify the threshold and the effective accumulated temperatures or that the data from the Yokohama population do not fit the Fukushima population or surrounding populations. Furthermore, the effective accumulated temperature for all stages is not accurate enough to predict stages and instars. Therefore, in the present study, we set the lower development threshold at 14.0 °C (see below), and the effective accumulated temperatures for each stage and instar were obtained based on our own field data.

The use of 14.0 °C as the threshold in the present study was based on the following reasons. First, the survival rate (percentage of the number of individuals normally eclosed) under the conditions of 15.0 °C has been reported to be more than 70% using the Kagoshima population of this species [60]. Since Kagoshima is located at the southernmost tip of mainland Japan, populations in Fukushima and its vicinity may be more tolerant to temperatures less than 15 °C. Second, in the same study, it has been reported that the survival rate was 0% at 10.0 °C [60]. Third, it has been reported that adult butterflies were observed until 8 December 2009 in Chichibu (Saitama Prefecture) [61]. The average value of the daily maximum temperature in the past 7 days from 8 December 2009 (i.e., average of 7 days) was 13.2 °C in Chichibu, suggesting that the lower threshold of adult activities is approximately 13.2 °C. Taken together, the threshold is likely in the range of 13.2–15.0 °C. Thus, we set the threshold at 14.0 °C, roughly at the middle point of the range.

A popular way to obtain the effective accumulated temperature (*k*) is to use a linear model; the daily temperature (*T*), the threshold (*t*), and the duration of *T*, usually in days (*D*), are related as follows: *k* = *D* (*T* − *t*) [62,63,64,65]. In the present study, we employed the daily maximum temperature for *T* instead of the daily average temperature. Operationally, the daily maximum temperatures above 14.0 °C were added together to yield the sum of the daily maximum temperatures as the effective accumulated temperatures, which is also known as the thermal constant. We reasoned that there was no need to maintain a greater temperature than the threshold temperature throughout a day for larval activities. If there was a period during which the temperature increased beyond the threshold during a day, it would be possible for the larvae to eat and grow. Since larvae of the pale grass blue butterfly may do just that, as suggested by the present field work, the daily maximum temperature may be more biologically relevant than the daily average temperature in the case of the pale grass blue butterfly. That is, the use of the maximum (instead of average) temperature is primarily based on our present findings on the butterfly’s life history. In addition, the average temperature data in the public database (see below) did not indicate any temperature variation within a day. This means that average temperatures below the lower development threshold (14.0 °C) do not ensure that larvae did not eat, because there may be hours of high temperatures above the threshold within a day. Therefore, we reasoned that the use of the average temperature data was not appropriate for this study.

The use of the threshold of 14.0 °C is based on the assumption that physiological activities in any stage respond to this threshold temperature. We also assumed that the threshold and the effective accumulated temperatures for growth were constant values in the seven representative localities, without any regional differences. We further assumed that these constant values were applicable to the past (and future) populations. Since we know the approximate dates of adult emergence in Fukushima and its vicinity in 2011 and because we also determined the prepupal and pupal durations (see below), the durations of the immature stages were traced back from the date of adult emergence in accordance with the temperature data of 2011 for the seven representative localities.

### 2.6. Rearing Experiment

In this study, immature stages in March 2011 were simulated based on possible durations of each stage and instar. Eclosion time points were first determined, and pupal, prepupal, and larval stages were assigned backwardly (see Results). Thus, it was necessary to obtain information on the durations (and the associated effective accumulated temperatures) of the prepupal and pupal stages and on pupal body size. To do so, larvae were collected from Fukushima and Kozaki in April 2019 and reared in our laboratory in accordance with standard rearing procedures [66,67]. Each larva was placed in a small Petri dish (10.0 mm height × 30.4 mm inner diameter), and the leaves of its host plant (collected in or around the university campus) were added every day or every other day at 15:00. Based on temperature records from the Japan Meteorological Agency (see below), the larvae were reared in incubators set at temperatures observed in Fukushima to simulate the temperature dynamics in the field. Mean values of daily average temperature (AT), daily minimum temperature (MinT), and daily maximum temperature (MaxT) for 3 days were obtained, and the cycle was set as follows: MinT (0:00–3:00 for 3 h), AT (3:00–12:00 for 9 h), MaxT (12:00–15:00 for 3 h), and AT (15:00–24:00 for 9 h). The temperature cycles were reset every 3 days. The mean times of sunrise and sunset for 3 days in Fukushima before starting the rearing experiment were 5:04 and 18:14, respectively, and differences from Kozaki were less than 5 min. Thus, we used the following light cycle throughout the rearing period: light period for 13 h (5:00–18:00) and dark period for 11 h (18:00–5:00). To determine the pupal period, 2 reared pupae from Fukushima were used (see Results). Two reared pupae from Kozaki were used only for size measurements. This rearing experiment began on 17 April 2019. Before this date, larvae were kept in the dark at 10 °C for 4 to 7 days. Since this temperature is below the lower development threshold (14.0 °C), we assumed that this period did not substantially affect the durations (and hence the effective accumulated temperatures) of the prepupal and pupal stages.

### 2.7. Public Climate Records

Temperature records were obtained from the website of the Japan Meteorological Agency (www.data.jma.go.jp/obd/stats/etrn/index.php). Due to the lack of exact locality data for each site except for Fukushima, the nearest available localities were used: Shiogama for Rifu, Onahama for Iwaki, Hitachi for Tokai-Mura, and Katori for Kozaki. Sunrise and sunset times were obtained from the Koyomi data of the National Astronomical Observatory of Japan (eco.mtk.nao.ac.jp/koyomi/dni). First-day observation data of animals in Fukushima City in 2019 were obtained from the Japan Meteorological Agency (www.data.jma.go.jp/sakura/data/index.html). This page included two butterflies, the Old World swallowtail butterfly, *Papilio machaon*, and the cabbage white butterfly, *P. rapae*. In Fukushima City in 2019, the former emerged on 16 May, and the latter emerged on 7 April.

### 2.8. Statistical Analyses

The statistical software R (The R Foundation for Statistical Computing, Vienna, Austria) was used to perform Spearman correlation analyses (cor.test(X, Y, method = “spearman”) after the Shapiro–Wilk test for normality (shapiro.test(X)). Linear regression analyses were performed using Microsoft Excel.

## 3. Results

### 3.1. Habitats and the Host Plants in the Field

**Figure 2:**
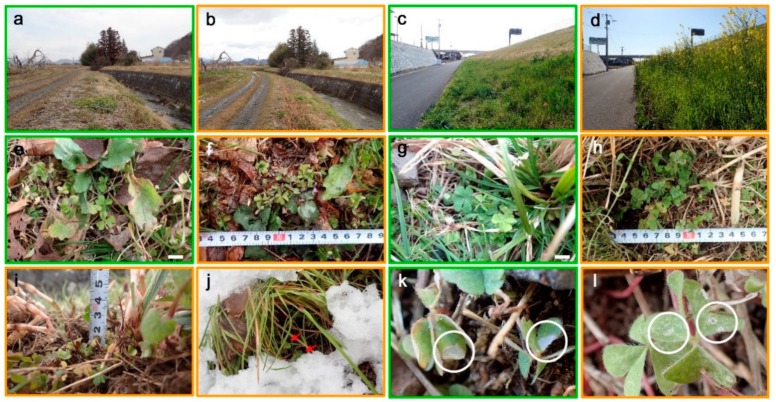
Habitats and the host plant *O. corniculata* in the field. Photographs taken in March are framed in green, and those taken in April are framed in yellow. The measuring tape (in **f**, **h**, and **i**) is in centimeters. (**a**–**d**) Research sites. (**a**) Fukushima in March 2019; (**b**) Fukushima in April 2019 (the same site as a); (**c**) Kozaki in March 2019; (**d**) Kozaki in April 2019 (the same site as c). (**e–l**) *O. corniculata*. (**e**) Rifu in March 2019. Scale bar, 10 mm; (**f**) Rifu in April 2019; (**g**) Iwaki in March 2019. Scale bar, 10 mm; (**h**) Iwaki in April 2019. The host plants are deep under dried grass; (**i**) Fukushima in April 2019; (**j**) Fallen snow in Rifu in April 2019. *Oxalis* is indicated by red arrows; (**k**) Feeding marks (circled) in Fukushima in March 2018; (**l**) Feeding marks (circled) in Iwaki in April 2019.

The habitats in Fukushima in March and April were mostly covered with dried grass, which may function as natural straw mulch (Figure 2a,b). In Kozaki, the southernmost locality in this study, there were already many plants with green leaves (Figure 2c,d). The host plant *O. corniculata* was very short but, importantly, it still had some leaves near the ground surface under dried grass or under snow (Figure 2e–j). On some leaves, the fresh feeding marks of young larvae characteristic of this butterfly species were discovered in March (Figure 2k), suggesting that the larvae eat during overwintering. Fresh feeding marks of young larvae were also discovered in April (Figure 2l), suggesting that larvae eat during or after overwintering.

### 3.2. Overwintering States in the Field

**Figure 3:**
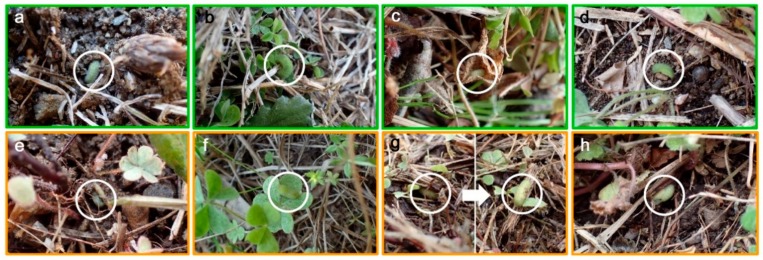
Larvae, prepupa, and pupa in the field (circled). Photographs taken in March are framed in green, and those taken in April are framed in yellow. (**a**–**f**) Larvae. (**a**) Fukushima in March 2018; (**b**) Iwaki in March 2018; (**c**) Rifu in March 2019, inside dried and curled *Oxalis* leaves; (**d**) Tokai in March 2019; (**e**) Fukushima in April 2019; (**f**) Kozaki in April 2019 on *Oxalis* leaves; (**g**) A prepupa in Fukushima in April 2019. The left panel shows the natural positioning, and the right panel shows a prepupa on dried straw after being removed; (**h**) A pupa in Iwaki in April 2019 on dried straw.

In total, we found 69 larvae in the three rounds of field surveys in situ (Figure 3a–f). Additionally, we found one prepupa and one pupa in April 2019 in situ (Figure 3g,h). In all three rounds of field work, the larvae were associated with the plant. They were mostly on the ground or on dried leaves and occasionally on the surface of leaves when discovered, but this variability could be because many larvae had fallen from their original positions due to the mechanical stimuli caused by field workers.

We discovered larvae of various instars (first to fourth instars) as follows (Table 1). In March 2018, we found 8 second-instar, 1 third-instar, and 1 fourth-instar larvae (10 larvae in total) in two localities. In March 2019, we found 1 first-instar, 18 second-instar, 7 third-instar, and 3 fourth-instar larvae in five localities (29 larvae in total). No other stages (eggs, prepupae, pupae, or adults) were found. It appeared that the larval stage was the overwintering stage, and there was no specific instar during overwintering (excluding the fifth instar). The most frequent was the second instar (26 larvae), followed by the third instar (8 larvae) and the fourth instar (4 larvae). Only 1 first-instar larva was found, but this is likely not because first-instar larvae did not exist, but because first-instar larvae were too small to be frequently visually detected by field workers.

In April 2019, when the overwintering period was over, we found various larvae in four localities as follows (Table 1): 10 second-instar, 12 third-instar, 7 fourth-instar, and 1 fifth-instar larvae (30 larvae in total). The discovery of the fifth instar, although represented by just 1 individual, is important because it was not found in March at all and because it suggests that larvae began to eat vigorously after the overwintering period to attain the fifth instar. It is likely that the fifth instar exists only after the overwintering period is over in this species, as mentioned in the literature [52,53,68]. The most frequent was the third instar (12 larvae), followed by the second instar (10 larvae) and the fourth instar (7 larvae). The frequency peak shifted from the second instar in March to the third instar in April, suggesting that the larvae grew during this one-month period.

In addition to these larvae, in April 2019, we found a prepupa and a pupa in situ. Both the prepupa and pupa were attached to a dried straw. Since neither prepupa nor pupa was found in March, this discovery in April suggests that the prepupal and pupal stages were not the overwintering stages. Instead, these individuals likely entered the prepupal and pupa stages in April after overwintering when it became warmer.

### 3.3. Body Sizes of the Overwintering Larva, Prepupa, and Pupa

The average body sizes of the overwintering larvae were calculated based on the body size data and the instar gaps (Table 2). The prepupal and pupal body sizes were also determined from field-caught and reared individuals. We considered that the body size data in Table 2 were applicable to the populations in the near past (and future), including 2010–2011 in Fukushima and its vicinity. In subsequent sections, we tried to specify a developmental stage or instar at the time of the Fukushima nuclear accident. Once specified, the body size of that immature stage or instar can be identified according to Table 2.

### 3.4. Effective Accumulated Temperatures

Thus far, we obtained the sum of the daily maximum temperature (above 14.0 °C), the body size, and the instar status for each larva. We calculated mean values for the daily maximum temperature for each instar (i.e., the mean accumulated temperatures) (mean ± SD) as follows: 234.6 DD (*n* = 1) for the first instar, 224.2 ± 128.8 DD (*n* = 36) for the second instar, 325.2 ± 137.7 DD (*n* = 20) for the third instar, 349.2 ± 165.6 DD (*n* = 11) for the fourth instar, and 489.4 DD (*n* = 1) for the fifth instar. These mean and standard deviation values are insightful to understand instars’ requirements for accumulated temperatures, but they do not indicate the effective accumulated temperatures (see below), which were needed for subsequent simulations.

**Figure 4:**
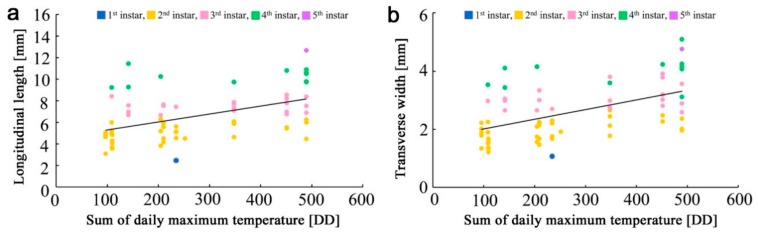
Scatter plots showing the relationships between larval body size and the sum of the daily maximum temperature. Colors of dots indicate larval instars. (**a**) Scatter plot for longitudinal length (larval length). A linear regression line is shown; (**b**) Scatter plot for transverse width (larval width). A linear regression line is shown.

The larval length and width were plotted against the sum of the daily maximum temperature (above 14.0 °C) (Figure 4a,b). There was a reasonable correlation between the sum of the daily maximum temperature and the larval length (*ρ* = 0.47, *p* = 5.7 × 10^−5^, *n* = 69). Similarly, there was a reasonable correlation between the sum of the daily maximum temperature and the larval width (*ρ* = 0.50, *p* = 1.1 × 10^−5^, *n* = 69). Both were statistically significant. A linear regression line was obtained for the larval length, which was *y* = 0.0073*x* + 4.6003 (*R*^2^ = 0.23), where *y* is the larval length and *x* is the sum of the daily maximum temperature. Likewise, a linear regression line was obtained for the larval width, which was *y* = 0.0033*x* + 1.6846 (*R*^2^ = 0.27).

Using the equation for the larval length and the size gaps between instars, we obtained the sum of the daily maximum temperatures above 14.0 °C required for specific instar periods (defined as the effective accumulated temperatures in the present study): 88.4 DD for the first instar, 461.0 DD for the second instar, 329.9 DD for the third instar, 350.4 DD for the fourth instar, and 166.2 DD for the fifth instar. We assumed that these values were applicable to the larvae of this species in the near past (and future), including 2010–2011 in Fukushima and its vicinity.

### 3.5. Rearing Experiment for Prepupal and Pupal Durations

We collected 8 individuals in Fukushima on 11 April 2019 and reared them to obtain information on the prepupal duration and pupal duration, referring to the actual daily temperatures in Fukushima (see Materials and Methods) (Table 3). Among them, 3 individuals did not reach the prepupal stage. Among the 5 individuals that reached the prepupal stage, 2 individuals reached the pupal stage. One individual spent 6 days in the prepupal stage and another spent only 2 days; thus, their average was 4 days. The reasons for this variability in the two individuals are not well understood. Assuming that an average prepupa spent 4 days from 27 April to 1 May 2019, the required sum of the maximum temperature for the prepupal stage was 92.7 DD.

Two pupae successfully eclosed to become adults. One pupa spent 18 days in the pupal stage, and the other pupa spent 23 days in the pupal stage. The average duration of the pupal stage was 20.5 days. Unfortunately, this was too long according to our rearing experience, and we did not use this result to specify pupal duration in the subsequent simulations. This result likely occurred because these two individuals were not completely healthy for various reasons; indeed, one of the adults showed relatively severe morphological abnormalities (deformation of wings, wrinkled wings, size asymmetry between the right and left wings, and deformation of antennae). It is also noteworthy that in the 8 reared individuals, the parasitization rate was 50%.

Nonetheless, it was necessary to specify the eclosion day for the overwintering states in 2011 for the sake of the simulation. Since we did not have any data to speculate on this point, we arbitrarily determined the expected pupal duration as 10 days (from 2 May to 12 May 2019) in accordance with our rearing experience. Fortuitously, the expected eclosion day of the pupae described above was similar to that of a different species, the Old World swallowtail butterfly, *P. machaon*. This butterfly has been considered to be a biological indicator for climate changes throughout Japan, including Fukushima. The public record indicated 12 May 2019 as the first day of observation of this butterfly in Fukushima. Accepting the pupal duration as 10 days, the required sum of the daily maximum temperature for the pupal stage was then determined to be 268.6 DD.

### 3.6. Eclosion Dates in 2011

After the effective accumulated temperatures (the thermal constants) for larval instars, prepupae, and pupae were determined, it was additionally required to determine the dates of pupal eclosion in 2011 for the sake of the simulation. In a previous study [41], we collected pale grass blue butterflies in May 2011 in Fukushima and its vicinity, which ensured that at the time of butterfly collection, pupal eclosion had occurred for at least some individuals (see Materials and Methods). Since we were able to collect some individuals that had likely eclosed freshly, as no wing damage was observed, and because collection was performed in the morning at some localities, we reasoned that a day before the collection date was a good approximation of the eclosion date. Thus, the eclosion dates in 2011 were determined as follows: 16 May for Motomiya, 15 May for Fukushima, 14 May for Hirono, Iwaki, and Takahagi, and 13 May for Mito and Tsukuba.

### 3.7. Estimates of Developmental Stages and Instars at the Time of the Fukushima Nuclear Accident

**Figure 5:**
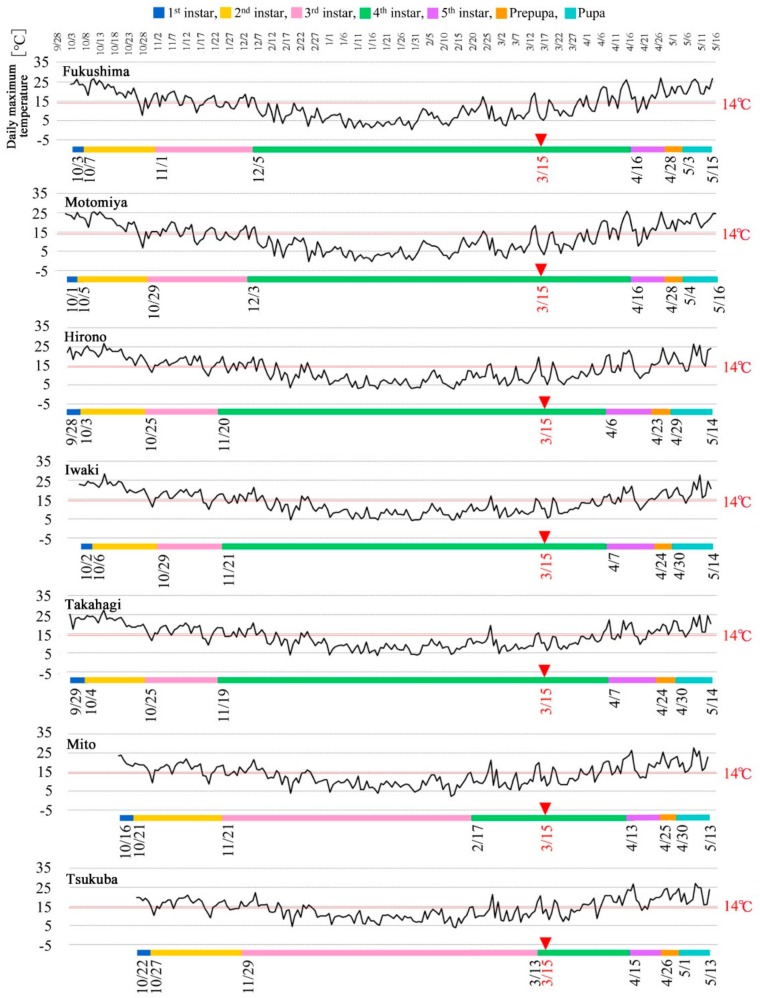
Timetables of developmental stages and larval instars in 2010–2011. Temporal changes in the daily maximum temperature are also shown above the timetables. The threshold temperature (14.0 °C) is indicated. The greatest emission date from the FDNPP (15 March 2011) is indicated by red arrowheads.

Using the eclosion dates determined above, the pupal effective accumulated temperature was first applied to the 2011 temperature data to determine the duration of the pupal period in the representative seven localities backwardly from the pupal eclosion dates. Similarly, the prepupal effective accumulated temperature was applied to the 2011 temperature data from the seven representative localities. For the larvae, the effective accumulated temperature for each instar (from the fifth to the first instar) was applied to the 2010–2011 temperature data to determine the instar periods. These calculations not only defined the number of days required for each stage and instar in 2010–2011 (Table 4) but also the exact starting dates of each stage in 2010–2011 (Table 5). These results were compiled to make timetables for the seven representative localities in 2010–2011 (Figure 5).

The instar that had the longest period was the fourth instar in Fukushima, Motomiya, Hirono, Iwaki, and Takahagi, but it was the third instar in Mito and Tsukuba (the southernmost localities among the seven representative localities). Mito and Tsukuba showed a different timetable pattern for the third and fourth instars from that shown by other localities, although other periods showed a similar pattern. The entire lifetime of an individual (without the adult stages) in Mito and Tsukuba was approximately 20 days shorter than that in other localities, which likely reflects late oviposition in October in Mito and Tsukuba. This is understandable because Mito and Tsukuba were warmer than the other localities. Despite these differences, the larvae in all seven representative localities were fourth instars on 15 March 2011, when the largest amount of emissions of artificial radionuclides occurred from the FDNPP.

### 3.8. Estimates of Body Sizes on 15 March 2011

The average size of the overwintering fourth-instar larvae was 10.22 mm (longitudinal length) × 3.98 mm (transverse width) (Table 2). Thus, larvae of these sizes were exposed to the maximum amounts of anthropogenic radionuclides on 15 March 2011. After that, these larvae became fifth instars, prepupae, pupae, and adults, during which they continued to be exposed. The cumulative exposed days from 15 March to the day of eclosion were as follows: 61 days in Fukushima, 62 days in Motomiya, 60 days in Hirono, Iwaki, and Takahagi, and 59 days in Mito and Tsukuba.

## 4. Discussion

We performed a series of field surveys to reveal the overwintering states of the pale grass blue butterfly in Fukushima and its vicinity in March 2018, March 2019, and April 2019. The periods of the field work were determined to reflect that the Fukushima nuclear accident occurred in March 2011. Although the field observations were physically demanding partly because of the small size of the objects, we discovered many overwintering larvae of various instars in situ. Since field discovery depends on the visual scanning ability of field workers, we cannot exclude other overwintering stages completely. Moreover, the smallest first-instar larvae were highly difficult to find (because they are too small for field workers to visually identify), and there may be a bias toward larger instars. However, with these reservations, it is likely that the larval stage is the major, if not the sole, overwintering stage of this species, at least in the surveyed localities. This is consistent with previous descriptions of the life history of the pale grass blue butterfly [51,52,53]. A related species, *Zizina emelina*, in Japan also overwinters as larvae [67]. In contrast, the present results are not consistent with the view that there is no specific overwintering stage [54]. It has been reported that in Kuno, Shizuoka City, all the life stages (eggs, larvae, pupae, and adults) of this butterfly were observed in December 1964, and that the larval stage (mainly the fourth instar) was the major overwintering stage in other regions in Shizuoka Prefecture [50]. Therefore, it is likely that the overwintering stage of this species in Japan depends on where observations are made. Thus, the present results are relevant to Fukushima and its vicinity, but not necessarily to other regions. In this sense, field surveys in other localities may be insightful to reveal regional differences in life history traits.

In the present study, we discovered various larval instars in March 2018 and 2019. This is true of warm localities (i.e., Iwaki, Tokai, and Kozaki), but only second-instar larvae were obtained from Rifu and Fukushima, the two coldest localities among those surveyed. This result may suggest that larval instar variation may be smaller in cold localities and larger in warm localities in March. Considering that there may be a bias toward larger larvae in the visual scanning process, the high abundance of second-instar larvae detected in this study is credible. In cold regions, young larvae cannot grow quickly and may stay at first or second instars for a relatively long time. Eggs, older larvae, prepupae, pupae, and adults may die out without successful overwintering in cold regions (see below for a discussion of the synchronization of first-voltine adults).

Since we found characteristic fresh feeding marks on leaves in March (also in April), it is likely that larvae consume leaves whenever possible during the winter season. On sunny days, larvae may be able to move and eat for a limited time because of the heat from the sun under the natural straw mulch, as we set the lower development threshold at 14.0 °C. We speculate that larval existence is dependent on the availability of host plant leaves during the winter season, which may be required for them to survive. From this perspective, the fact that the second-instar larvae were most frequent in March in colder localities makes sense. Early-instar larvae require only a small amount of leaves, which may be available in winter. The distribution of the instars in April 2019 was skewed to the larger side in comparison to that in March 2019, which suggests that the larvae might have grown during this one-month period. This interpretation may be correct, considering that no fifth-instar larvae were found in March, although visual discovery may be biased toward larger individuals. Unfortunately, we could not perform field work in April 2018, and thus we cannot present confirmatory data at this point.

This line of discussion is consistent with the view presented in a Japanese book, which mentioned that fourth-instar larvae cannot survive due to a lack of sufficient amounts of fresh host plant leaves, and that second-instar and third-instar larvae can survive and grow slowly by eating the small amounts of fresh leaves available in winter [68]. The book also mentioned that these second and third instars become fourth instars at the end of winter and then become fifth instars in early spring [68]. Although no data were presented in the book, our results from Fukushima and its associated localities are consistent with this view. This view is also expressed in a few lepidopterological books [51,52]. In a similar species, *Z. emelina*, eating and molting have been observed during the overwintering period [67]. On the other hand, the present results are not consistent with other descriptions in the literature: larvae do not eat at all during the winter season [50]. The discrepancy between the results from the present study and these descriptions is not well understood, but may reflect differences in the localities where the observations were made. Further systematic studies are required to clarify this point.

If our view of the overwintering states is correct, how the host plant is maintained in winter is one of the important factors affecting the survival of the pale grass blue butterfly. Natural straw mulch may be important for leaves and larvae to survive. This may not be surprising from an agricultural perspective. Straw mulch has been known to increase the ground surface temperature and prevent water in the soil from evaporating [69,70].

It has been known that the first-voltine adults (the spring form) are larger in body size than the second-voltine (and subsequent) adults in the pale grass blue butterfly in Japan. This is because the first-voltine overwintering larvae reach fifth instar, whereas others end with fourth instar [52,53], which is consistent with the results of the present study. The variable number of instars within a species is likely widespread among insects, and it is generally observed among Lepidoptera that the number of instars increases under adverse conditions [71]. An additional molt may be stationary (without growth) or even regressive in some species [71], but larvae of the pale grass blue butterfly appear to grow with an additional molt after overwintering.

The large fifth-instar larvae likely require a relatively large amount of leaves. It is reasonable that the relatively rapid growth of leaves in April may help the larvae eat them vigorously. Although there are no numerical data, our experience indicates that the number of first-voltine adults is relatively small at the population level and that this butterfly increases its number of individuals toward the fall season. This means that a large proportion of overwintering individuals indeed die in winter. This further means that the emergence of adults at a given site may be synchronized in the first-voltine populations. More broadly, the endpoint (eclosion) did not vary much among the seven representative localities in our field surveys in 2011–2013 [41,42]. The death of overwintering individuals except for relatively young larvae may contribute to this synchronization of first-voltine adults in May, which may be more strict in relatively cold regions. Such death may be caused by low temperatures that lead to a lack of host plant leaves, failure of molting, pupation, and eclosion, or parasitism. In our rearing experiment, the parasitization rate was 50%. It appears that the parasitization rate varies depending on space and time in lycaenid butterflies [72,73]. Larvae may be protected from parasitoids by attending ants [73], which may not be available in winter.

Based on the results that were obtained from the field work and laboratory rearing experiment, we simulated the overwintering states in seven representative populations, including that in Fukushima in 2011. To do so, we assumed that the larval temperature–size relationship is applicable to past populations. We also assumed that the sum of the daily maximum temperature at the threshold of 14.0 °C was important for physiological activities. This threshold may be conservative, and there is a possibility that the Fukushima populations, especially at the larval stage, are more resistant to colder temperatures. However, we obtained reasonable correlation coefficients with very small *p*-values for both the larval length and width, justifying our assumptions for the purpose of the simulations.

The effective accumulated temperatures based on the body-size regression equation were larger than or similar to the mean accumulated temperatures in the second, third, and fourth instars, as theoretically expected, whereas those of the first and fifth instars were not, indicating that there is a room for improvement. Standard deviation values of the mean accumulated temperatures showed that these data likely had relatively high uncertainty. The *R*^2^ value of the regression equation used for the determination of the effective accumulated temperatures for each instar was also relatively low. Thus, the durations and dates of immature stages and instars should be considered as representative values for the sake of simulations. As long as these points are understood, we believe that our data and results are invaluable for the simulations.

The effective accumulated temperatures were the smallest for the first instars and the highest for the second instars. The second instars may require more time and energy to grow, which may be related to the fact that the first instars do not appear to have a functional nectary organ to attract ants, but such an organ appears to be functional in the second and subsequent instars in this and other *Zizeeria* and *Zizina* species [74]. The fifth instar was associated with a relatively small value of the effective accumulated temperature, but this small value does not necessarily mean that a small amount of plant material was consumed.

The estimation of the effective accumulated temperatures for the prepupal and pupal stages is not satisfactory in the present study, considering that only a relatively small number of individuals were successfully reared and that the eclosion date was arbitrarily determined. Moreover, the eclosion timing of pupae may be influenced not only by the effective accumulated temperatures but also by exposures to temperatures above (or below) a threshold and to long day length. These factors were not considered in the present study because of a lack of information regarding these points, but they may also contribute to the synchronization of first-voltine adults. The effective accumulated temperatures for larval instars were obtained from field-based observations, which may be compared with those obtained from laboratory-based rearing observations in the future. The validity of the threshold of 14.0 °C may also be examined in laboratory-reared individuals.

We presented timetables for the life stages of the pale grass blue butterfly during the overwintering states in Fukushima and its vicinity in 2010–2011. These timetables are important but somewhat misleading in that they are applicable only to typical individuals. In reality, there would be various stages and instars especially in early winter, because ovipositioning dates in September and October 2010 in the field likely vary. As shown by the field work, there may be at least a few different larval instars at a given time point. It is also to be noted that the endpoints (eclosion dates) were first determined in this simulation, but in reality, they would also vary.

Nonetheless, the timetables are helpful to understand possible convergence toward similar eclosion dates among the representative seven localities. Among the seven timetables that are presented, those for the two southernmost populations, Mito and Tsukuba, differed from those for the other five northern populations in terms of the durations of the second and third instars. In these two populations, the major overwintering instar is the third instar, whereas in the other five populations, it is the fourth instar. The northern range margin populations may be further different. This variability in instars is interesting, considering that this butterfly is expanding its northern range margin [75].

According to our simulations, the pale grass blue butterfly on 15 March 2011 was fourth-instar larvae. In the field work in March 2018 and 2019, fourth-instar larvae were relatively rare, and younger instars were more frequent. This difference likely comes from the yearly difference in temperatures. Relatively large larval size and possibly high metabolic activities in fourth instars at the time of the Fukushima nuclear accident might have contributed to an increase in sensitivity to radioactive exposures, albeit a limited one.

An early series of studies on the pale grass blue butterfly in Fukushima focused on clarifying the causal contributions of the Fukushima nuclear accident to the butterfly without paying much attention to the conventional mechanisms of radiation-induced biological effects. The mechanistic aspects of the biological effects are now a focus of our research. The significance of the present study is that the overwintering developmental states of the pale grass blue butterfly in Fukushima and its vicinity were successfully simulated and that the immature state on 15 March 2011 was successfully specified. We concluded that the butterfly was in the fourth instar on 15 March 2011. This information is required for accurate dosimetric estimates of the absorbed radiation doses, which will make us understand the relationship between field absorbed doses and their biological impacts on the pale grass blue butterfly. Additionally, dosimetric calculations likely contribute to the understanding of transgenerational effects [47,76,77] and the field–laboratory paradox of the biological effects of radioactive pollution, at least in this butterfly [78,79,80,81].

Evolutionary speaking, the origin of the genus *Zizeeria* may be in Africa, although no solid evidence exists to our knowledge. The African grass blue butterfly, *Zizeeria knysna*, consumes several different host plants, including *O. corniculata*, with which the butterfly can be reared successfully [82]. On the other hand, the dark grass blue butterfly, *Zizeeria karsandra*, which is distributed in Southeast Asia, does not consume *O. corniculata*. A recent study suggested that the origin of *O. corniculata* is in Southeast Asia [83]. The ancestor of the pale grass blue butterfly might have become monophagous in Asia where *O. corniculata* is distributed widely, resulting in the differentiation into the present species. Then, the pale grass blue butterfly expanded its distribution range to the north, up to Japan, with the northern expansion of its host plant, where the overwintering strategies evolved gradually in response to the winter conditions in particular environments. Since the northern range margin is now expanding to the north [75], it is interesting to know whether the overwintering strategies may become more elaborate in the northern populations.

## 5. Conclusions

This study revealed that the pale grass blue butterfly overwintered as larvae of various instars (but not as the fifth instar and not as other stages) in Fukushima and its vicinity in March 2018 and 2019. The larvae likely consume leaves during the winter season when the temperature is below the lower development threshold, which is defined here as 14.0 °C. We deduced that the butterfly was typically present as fourth-instar larvae in Fukushima and its vicinity at the time of the Fukushima nuclear accident in March 2011. This study paves the way for estimating absorbed radiation doses of the first-generation butterflies that expressed adverse transgenerational effects in subsequent generations.

## Figures and Tables

**Table 1:** Localities of field work and the number of individuals found in situ.

Locality	Date	Number of Found Individuals							
		First Instar	Second Instar	Third Instar	Fourth Instar	Fifth Instar	Prepupa	Pupa	Total
Rifu	10 March 2019	0	6	0	0	0	0	0	6
	10 April 2019	0	1	0	0	0	0	0	1
Fukushima	10 March 2018	0	5	0	0	0	0	0	5
	12 March 2019	1	4	1	0	0	0	0	6
	11 April 2019	0	2	5	1	0	1	0	9
Iwaki	12 March 2018	0	3	1	1	0	0	0	5
	9 March 2019	0	3	1	1	0	0	0	5
	12 April 2019	0	4	4	1	0	0	1	10
Tokai	12 March 2019	0	0	3	2	0	0	0	5
Kozaki	13 March 2019	0	5	2	0	0	0	0	7
	13 April 2019	0	3	3	5	1	0	0	12
	Total	1	36	20	11	1	1	1	71

**Table 2:** Body size of larvae, prepupa, and pupae.

	Longitudinal Length [mm]			Transverse Width [mm]			*n*
	Mean ± SD	Min.	Max.	Mean ± SD	Min.	Max.	
First instar	2.48	NA	NA	1.08	NA	NA	1
Second instar	4.98 ± 0.83	3.13	6.32	1.92 ± 0.36	1.22	2.66	36
Third instar	7.50 ± 0.58	6.67	8.56	3.07 ± 0.40	2.60	3.92	20
Fourth instar	10.22 ± 0.68	9.24	11.46	3.98 ± 0.51	3.12	5.10	11
Fifth instar	12.67	NA	NA	4.76	NA	NA	1
Prepupa	9.88	NA	NA	4.00	NA	NA	1
Pupa	9.38 ± 0.41	8.64	9.71	3.94 ± 0.22	3.55	4.19	5 *

* One individual found in the field in April and four individuals reared from larvae. NA: not applicable.

**Table 3:** Rearing of larvae from Fukushima.

Individual No.	Starting Instar	Prepupa	Pupa	Adult	Death	Parasitoid
1	Second instar	-	-	-	18 April	NA
2	Second instar	-	-	-	23 April	Wasp
3	Second instar	-	-	-	18 April	NA
4	Third instar	30 April	6 May	24 May	NA	NA
5	Third instar	27 April	29 April	22 May	NA	NA
6	Third instar	5 May	-	-	9 May	Fly
7	Third instar	30 April	-	-	6 May	Fly
8	Fifth instar	19 April	-	-	28 April	Fly

Note: Dates at the beginning of stages are shown. A dash indicates that individuals did not reach that stage. NA indicates “not applicable”. Only individuals No. 4 and No. 5 reached the adult stage. No. 4 was morphologically normal, and No. 5 showed relatively severe morphological abnormalities.

**Table 4:** Number of days required for each instar and stage in 2010–2011.

	First Instar	Second Instar	Third Instar	Fourth Instar	Fifth Instar	Prepupa	Pupa	Total
Fukushima	4	25	34	132	12	5	13	225
Motomiya	4	24	35	134	12	6	13	228
Hirono	5	22	26	137	17	6	16	229
Iwaki	4	23	23	137	17	6	15	225
Takahagi	5	21	25	139	17	6	15	228
Mito	5	31	88	55	12	5	14	210
Tsukuba	5	33	104	33	11	5	13	204

**Table 5:** Starting dates of each instar and stage in 2010–2011.

Locality	First Instar	Second Instar	Third Instar	Fourth Instar	Fifth Instar	Prepupa	Pupa	Adult
Fukushima	3 October	7 October	1 November	5 December	16 April	28 April	3 May	15 May
Motomiya	1 October	5 October	29 October	3 December	16 April	28 April	4 May	16 May
Hirono	28 September	3 October	25 October	20 November	6 April	23 April	29 April	14 May
Iwaki	2 October	6 October	29 October	21 November	7 April	24 April	30 April	14 May
Takahagi	29 September	4 October	25 October	19 November	7 April	24 April	30 April	14 May
Mito	16 October	21 October	21 November	17 Feburary	13 April	25 April	30 April	13 May
Tsukuba	22 October	27 October	29 November	13 March	15 April	26 April	1 May	13 May
